# Calibrating 3D Scanner in the Coordinate System of Optical Tracker for Image-To-Patient Registration

**DOI:** 10.3389/fnbot.2021.636772

**Published:** 2021-05-14

**Authors:** Wenjie Li, Jingfan Fan, Shaowen Li, Zhaorui Tian, Zhao Zheng, Danni Ai, Hong Song, Jian Yang

**Affiliations:** ^1^Beijing Engineering Research Center of Mixed Reality and Advanced Display, School of Optics and Photonics, Beijing Institute of Technology, Beijing, China; ^2^Ariemedi Medical Technology (Beijing) CO., LTD., Beijing, China; ^3^School of Computer Science and Technology, Beijing Institute of Technology, Beijing, China

**Keywords:** 3D scanner, calibration, tracker, image-guided surgery, image-to-patient registration

## Abstract

Three-dimensional scanners have been widely applied in image-guided surgery (IGS) given its potential to solve the image-to-patient registration problem. How to perform a reliable calibration between a 3D scanner and an external tracker is especially important for these applications. This study proposes a novel method for calibrating the extrinsic parameters of a 3D scanner in the coordinate system of an optical tracker. We bound an optical marker to a 3D scanner and designed a specified 3D benchmark for calibration. We then proposed a two-step calibration method based on the pointset registration technique and nonlinear optimization algorithm to obtain the extrinsic matrix of the 3D scanner. We applied repeat scan registration error (RSRE) as the cost function in the optimization process. Subsequently, we evaluated the performance of the proposed method on a recaptured verification dataset through RSRE and Chamfer distance (CD). In comparison with the calibration method based on 2D checkerboard, the proposed method achieved a lower RSRE (1.73 mm vs. 2.10, 1.94, and 1.83 mm) and CD (2.83 mm vs. 3.98, 3.46, and 3.17 mm). We also constructed a surgical navigation system to further explore the application of the tracked 3D scanner in image-to-patient registration. We conducted a phantom study to verify the accuracy of the proposed method and analyze the relationship between the calibration accuracy and the target registration error (TRE). The proposed scanner-based image-to-patient registration method was also compared with the fiducial-based method, and TRE and operation time (OT) were used to evaluate the registration results. The proposed registration method achieved an improved registration efficiency (50.72 ± 6.04 vs. 212.97 ± 15.91 s in the head phantom study). Although the TRE of the proposed registration method met the clinical requirements, its accuracy was lower than that of the fiducial-based registration method (1.79 ± 0.17 mm vs. 0.92 ± 0.16 mm in the head phantom study). We summarized and analyzed the limitations of the scanner-based image-to-patient registration method and discussed its possible development.

## 1. Introduction

The rapid development of 3D scanning devices has introduced the possibility of acquiring high-quality 3D models within seconds. Three-dimensional scanners have been widely applied in image-guided surgery (IGS) given their potential to solve the image-to-patient registration problem (Cao et al., 2008; Fan et al., 2014, 2020), which directly affects the positioning accuracy of surgical navigation systems. As the core step in IGS, image-to-patient registration has attracted considerable research attention worldwide (Fitzpatrick et al., 2002; Gerber et al., 2013; Chu et al., 2017; Kim and Kazanzides, 2017).

In early IGS systems, image-to-patient registration was mostly based on artificial fiducials, which was called fiducial-based registration. The fiducial-based registration method was first applied to the clinic by Roberts et al. (1986), and several special fiducials were attached to the skin to align the CT image and the operating microscope. Maurer et al. (1997) used implantable fiducials in IGS, and the clinical results showed that the target registration error (TRE) ranged from 0.5 to 0.6 mm, reaching a submillimeter accuracy. Kim and Kazanzides (2017) proposed a fiducial-based registration framework that uses fiducials with a specific shape to position in CT images, thereby reducing the positioning error introduced by the operator. However, the fiducial-based registration method is limited by its disadvantages, such as complicated operation, high time cost, trauma, and potential hazard in contact.

In recent years, the image-to-patient registration method has evolved from fiducial-based registration to surface-based registration (Lathrop et al., 2010; Simpson et al., 2012; Ji et al., 2015; Fan et al., 2016) to achieve a fast, nonfiducial, and noninvasive image-to-patient registration. A series of surface-based image-to-patient registration methods have been proposed and have quickly become a research hotspot. Simpson et al. (2012) compared several tools for intraoperative surface acquisition, including tracked laser range scanners (LRS), tracked pointers, and tracked conoscopic holography sensors, and found that the LRS-based facial spatial digitization method performs best. Generally, a tracked marker needs to be fixed on the 3D scanner, and some specific calibration procedures are then conducted to establish a coordinate relationship between the 3D scanner and the external tracker, such as hand-eye calibration (Tsai and Lenz, 1989; Heller et al., 2016; Wan and Song, 2020).

However, the application of 3D scanner-based image-to-patient registration still has many limitations. A summary of image-to-patient registration methods in IGS (Willems et al., 2001; Schicho et al., 2007; Woerdeman et al., 2007; Grauvogel et al., 2010; Soteriou et al., 2016; Zhao et al., 2018) reveals that conventional fiducial-based registration methods always outperform surface-based ones. Eggers et al. (2006) revealed that the residual rotational error is the most significant factor for the deviation of surface-based image-to-patient registration. Therefore, eliminating the residual rotational error in the extrinsic parameters of a 3D scanner is critical to improve the performance of 3D scanner-based image-to-patient registration.

In this study, a dedicated 3D benchmark and a novel calibration method were proposed to calibrate the extrinsic parameters of a 3D scanner in the coordinate system of an optical tracker. The main contributions of this study are summarized as follows.

1. A 3D benchmark for calibrating a 3D scanner was designed to compensate for the large residual errors in 2D checkerboard-based 3D scanner calibration tasks. The designed benchmark can also be applied to various calibration scenarios of different types of 3D scanner.
2. A two-step calibration method was proposed to calibrate the extrinsic parameters of the 3D scanner based on pointset registration technique and nonlinear optimization algorithms. In comparison with the conventional hand-eye calibration method based on 2D checkerboard, the proposed method showed better performance in verification experiments.
3. A complete 3D scanner-based image-to-patient registration framework was proposed, and the proposed two-step calibration method was applied to achieve the image-to-patient registration procedure.

The rest of this paper is organized as follows. In section 2, we reviewed the conventional camera calibration method based on 2D checkerboard. In section 3, we explained our calibration method in detail. In section 4, we validated and compared our proposed approach with state-of-the-art methods. We summarized those factors that restrict the development of the scanner-based method for image-to-patient registration and then described the possible future research directions.

## 2. Related Works

In this section, we initially summarized the related works on 2D camera calibration and then reviewed some works related to estimating the extrinsic parameters of 3D scanners.

### 2.1. 2D Camera Calibration

Camera calibration is a fundamental task in the field of computer vision. Through the calibration process, the mapping relationship between the 3D world and the 2D image captured by the camera is established, thereby guiding the computer to recognize the entire real world. In the camera calibration task, the camera is usually simplified as a pinhole model. On the basis of this model, the intrinsic matrix (K) of the camera can be formulated as

(1) $$\begin{array}{l} K=\left[\begin{array}{ccc} f_{x} & 0 & u_{0}\\ 0 & f_{y} & v_{0}\\ 0 & 0 & 1 \end{array}\right] \end{array}$$

where *u*_0_ and *v*_0_ are the principal points, and *f*_*x*_ and *f*_*y*_ are the focal lengths. For 2D camera calibration methods, a calibration benchmark with specific geometric properties that are easy to identify and extract is often used. Zhang (2000) proposed a 2D calibration benchmark with a checkerboard image. The corresponding calibration method requires the camera to take at least three images containing the checkerboard image at different positions. At the same time, a certain number of fixed corners of the checkerboard is taken to calculate the intrinsic and extrinsic parameters in the world coordinate system. This method has been widely used in academic research and industrial fields due to its simplicity of operation and high precision.

### 2.2. Calibrating the 2D Camera in an External Coordinate System

With the development of robotic technology, calibrating the camera in the external coordinate system (robot workspace) is necessary to enable the robot to acquire and understand the appropriate information about its workspace. In the industrial robot system, the relative transformation between the coordinate system of a camera and that of a robot must be initially determined, and the robot can then perform its specific tasks autonomously. This situation is the well-known hand-eye calibration problem.

**Figure 1:**
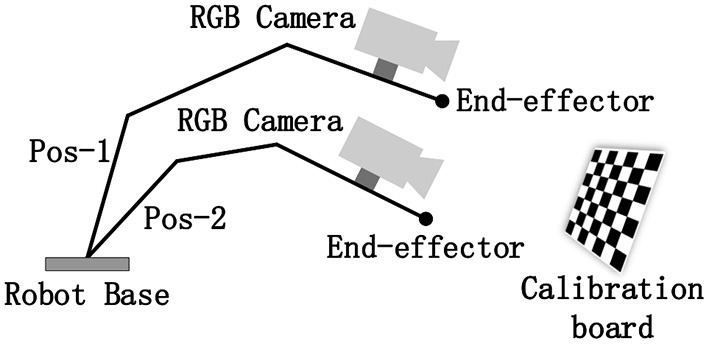
Schematic of the hand–eye calibration problem.

The hand-eye calibration problem (Tsai, 1987; Daniilidis and K., 1999; Heller et al., 2016) can be clearly described by the mathematical formula *AX* = *XB*, where *X* is the unknown matrix to be estimated. Solving this problem usually requires capturing data in multiple positions. Afterward, a series of equations are established to solve the final transformation matrix (also called the extrinsic matrix), as shown in Figure 1.

### 2.3. Calibrating the 3D Scanner in an External Coordinate System

Other than the 2D images projected by a camera, a 3D scanner can capture the 3D geometric shapes of objects. A high-quality 3D scanner provides manifold solutions for diverse fields, such as computer-assisted surgery and robotic system with 3D vision. The general structure of the structured-light-based 3D scanner usually comprises a binocular camera for 3D reconstruction and an RGB camera for capturing texture images, and the coordinate mapping between the RGB and binocular cameras requires an additional transformation $T_{RGB}^{Scanner}$. In this case, similar to 2D camera calibration methods, the extrinsic parameters of 3D scanners can be calibrated using a 2D checkerboard by considering $T_{RGB}^{Scanner}$.

**Figure 2:**
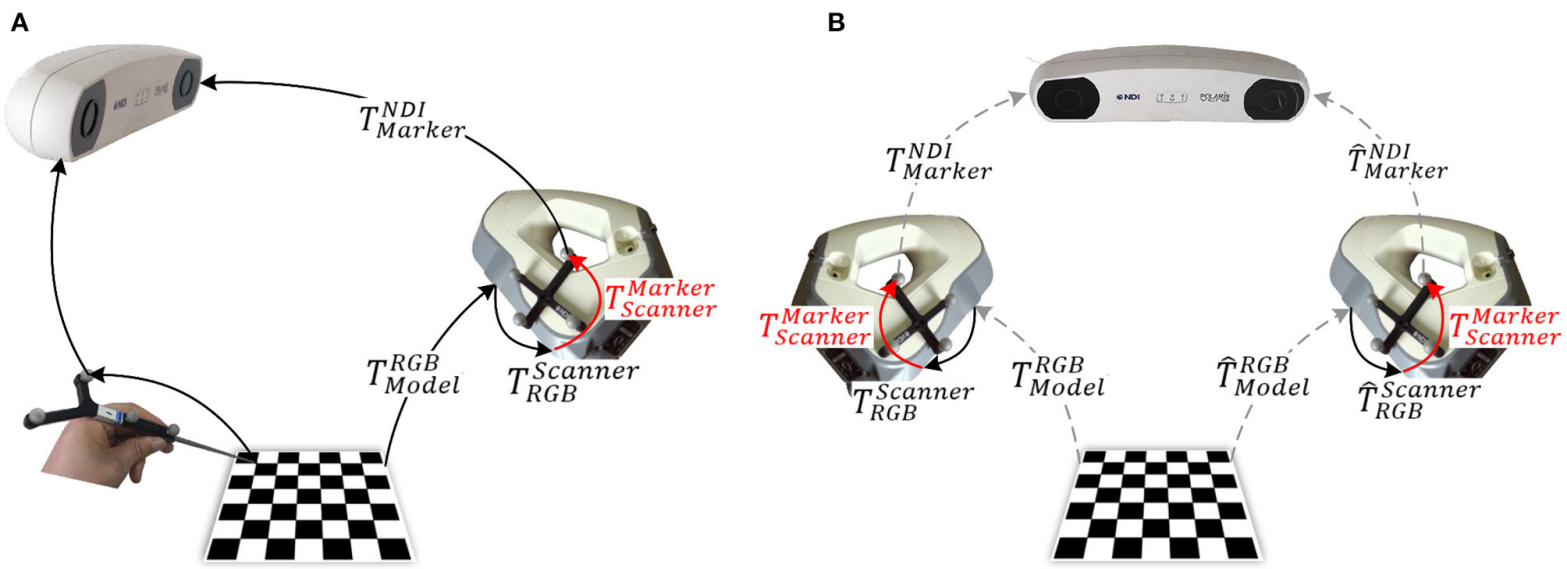
Methods for 3D scanner calibration based on a 2D checkerboard: **(A)** closed-form solution (Pick-2D): pick points using a tracked pointer; and **(B)** numerical solution (Hand–Eye-2D): based on hand–eye calibration algorithm.

Figure 2 shows two commonly used methods for calibrating the 3D scanner based on a 2D checkerboard. Except for special instructions, the coordinate system of the external tracker is regarded as the world coordinate system throughout the rest of the study. Figure 2A illustrates a closed-form solution for 3D scanner calibration using a tracked pointer. The 3D world coordinates of the corners in the 2D checkerboard are picked by the tracked pointer. At the same time, the corresponding corners are extracted from the captured RGB images. Combined with the known $T_{RGB}^{Scanner}$, the relationship between the captured corners in different coordinates forms a closed loop. Then, the optimal target transformation $T_{Scanner}^{Marker}$ can be solved by SVD algorithm easily. Figure 2B illustrates a numerical solution, where the corners acquired in the RGB images are mapped to the pointsets in the 3D space for calibration. The subsequent operation is similar to the hand-eye calibration method of the RGB camera mentioned above, where multiple sets of data need to be collected in different positions and a series of equations are constructed to estimate the final transformation.

However, given that $T_{RGB}^{Scanner}$ is an approximation with residual error. This residual error will accumulate in multiple spatial transformations and eventually lead to the increase in positioning errors, which will be evaluated in the experiments section. Although various applications based on the tracked 3D scanner have been proposed (Pheiffer et al., 2012; Fan Y. et al., 2017), 3D scanner calibration in the external coordinate system requires a better solution.

**Figure 3:**
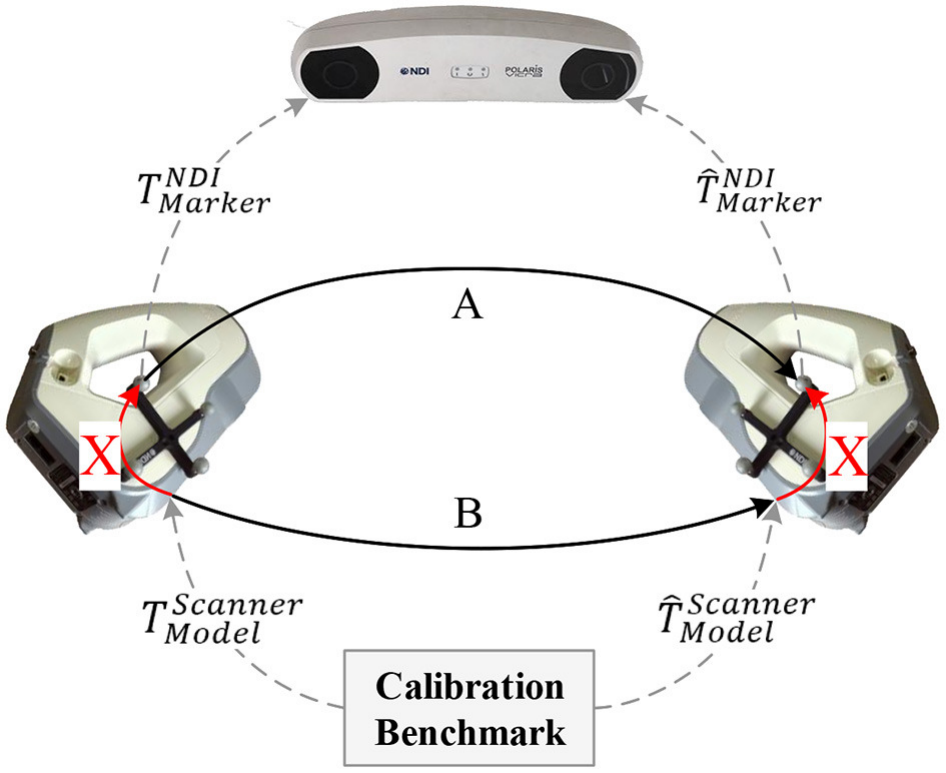
Proposed calibration method for the 3D scanner.

Figure 3 illustrates a schematic of the proposed method for calibrating the extrinsic parameters of the 3D scanner. Four related coordinate systems are the optical tracker {NDI}, optical marker {Marker}, 3D scanner {Scanner}, and calibration benchmark {Model}. By sorting out the coordinate transformation relationship shown in the figure, we obtain the hand-eye calibration equation in general form, *AX* = *XB*, as shown as follows:

(2) $$\begin{array}{l} ({\hat{T}}_{Marker}^{NDI}{)}^{-1}T_{Marker}^{NDI}T_{Scanner}^{Marker}={\hat{T}}_{Scanner}^{Marker}{\hat{T}}_{Model}^{Scanner}(T_{Model}^{Scanner}{)}^{-1} \end{array}$$

where *X* ($T_{Scanner}^{Marker}$) is the unknown matrix to be estimated, and matrices A and B can be obtained by performing additional calculations. Several methods for solving the AX = XB equation have been proposed in academic and engineering practice (Tsai and Lenz, 1989; Daniilidis and K., 1999; Heller et al., 2016). Given the particularity of 3D scanner calibration, this study focuses on three parts, namely, (1) the calibration benchmark design; (2) the solution of the relative parameters in the equation, including *A*, *B*, and *X*; and (3) the global optimization strategy for fine-tuning the target transformation *X*.

## 3. Methods

In this study, we calibrated the extrinsic parameters of the tracked 3D scanner. First, we designed and printed a specified 3D benchmark through 3D printing technology. On this basis, we constructed a novel calibration framework via pointset registration to calibrate the extrinsic matrix of the 3D scanner. Afterward, we proposed a global optimization strategy based on the nonlinear optimization technique to fine-tune the obtained extrinsic matrix. We eventually constructed a surgical navigation system and used the calibrated 3D scanner to achieve the image-to-patient registration process.

### 3.1. 3D Benchmark Design

Given the particularity of the 3D scanner calibration, a specific benchmark that meets the following design principles should be designed: (1) the discernibility of the benchmark should be guaranteed to ensure that the captured data at different positions and orientations can be aligned accurately; (2) the 3D features of the benchmark should be sufficient to ensure the accuracy of pointset registration; and (3) the integrity of the visible surface of the benchmark at different positions and orientations should be guaranteed to capture as much surface data as possible.

**Figure 4:**
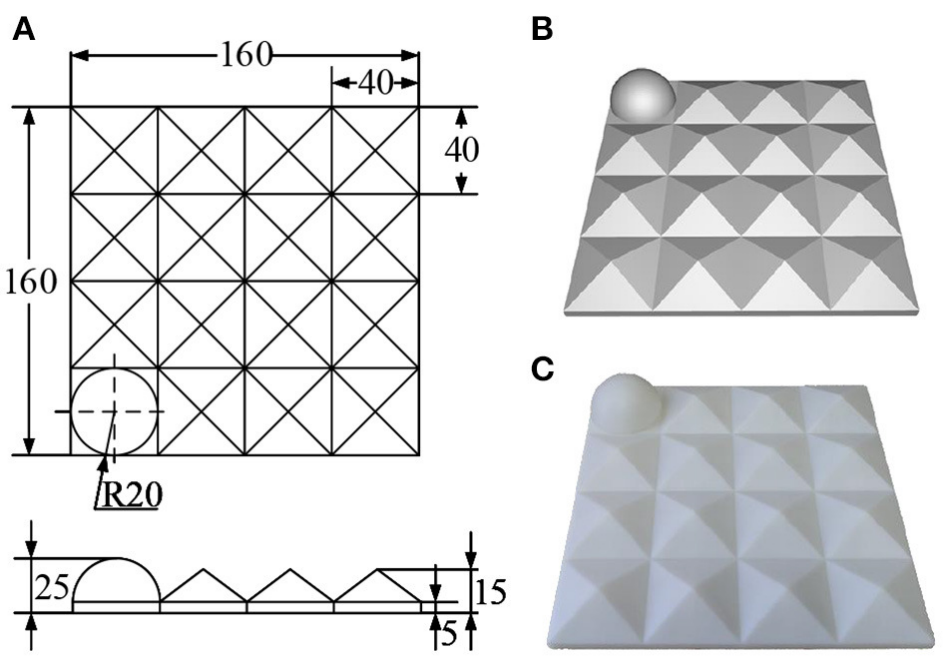
Designed 3D calibration benchmark: **(A)** engineering drawing (mm); **(B)** rendering drawing; and **(C)** physical drawing.

On the basis of these design principles, a specific benchmark for calibrating the extrinsic parameters of the 3D scanner was designed and printed through 3D printing technology. The printing error was controlled within 0.2 mm as shown in Figure 4.

The designed benchmark comprises 4 × 4 3D submodels. Except for the hemisphere (r = 20 mm) located at the corner for determining direction, the rest is filled with pentahedrons (l = 40 mm, h = 15 mm). This design aims to ensure that most of the surface of the benchmark can be captured while maintaining as many 3D features as possible. Moreover, this design can improve the ability of the captured data to describe the 3D space and the calibration accuracy of the 3D scanner to a certain extent. The designed 3D benchmark can also be used for the calibration tasks of the 3D scanner in various other scenarios.

### 3.2. Determining the Equation Parameters

Using the calibration benchmark mentioned above, we comprehensively described the calculation of the parameters in the proposed calibration framework. Matrix A represents the relative transformation of the optical marker when scanning in different positions and orientations, which can be calculated directly from the tracking data, and its accuracy is determined by the positioning error of the tracker. The positioning error of the optical tracker (Polaris VEGA, Northern Digital Inc., Canada) used in this study is 0.12 mm, which is extremely small and hence will not be discussed.

Matrix *B* represents the relative transformation of the 3D scanner in space. To estimate this matrix, we initially fixed the designed benchmark in the world coordinate system. Then the surface of the benchmark was captured by the 3D scanner in different positions and orientations. Assuming that *p*_*m*_ is a random point in the coordinate system of the benchmark, the corresponding point in the pointset captured by the scanner shown in Figure 3 can then be formulated as

(3) $$\begin{array}{l} \{\begin{array}{c} p_{m}^{\prime }=T_{Model}^{Scanner}p_{m}\\ {\hat{p}}_{m}^{\prime }={\hat{T}}_{Model}^{Scanner}p_{m} \end{array} \end{array}$$

Eliminating the common parameters *p*_*m*_ in Equation (3) yields

(4) $$\begin{array}{l} {\hat{p}}_{m}^{\prime }={\hat{T}}_{Model}^{Scanner}(T_{Model}^{Scanner}{)}^{-1}p_{m}^{\prime }=Bp_{m}^{\prime } \end{array}$$

According to Equation (4), matrix *B* in Figure 3 is the spatial transformation between the captured pointsets. The matching between pointsets was achieved by the iterative closest point (ICP) algorithm (Besl and Mckay, 1992), which basic principle is to iteratively find a transformation that best aligns two pointsets. This algorithm achieves this principle by minimizing the following alignment errors

(5) $$\begin{array}{l} E_{reg}\left(R,t\right)=\frac{1}{n}\overset{n}{\underset{i=1}{\sum }}\|q_{i}-\left(Rq_{i}+t\right){\|}^{2} \end{array}$$

where *R* and *t* represent the rotation matrix and translation vector that minimize the overall error between the two pointsets, respectively; and *p*_*i*_ and *q*_*i*_ represent the *i*-th point in these pointsets. Through pointset registration, the relative transformation matrix of the scanner in different positions and orientations could be estimated. By capturing multiple sets of data in different positions and orientations, we could construct a series of equations in the form of *AX* = *XB*. The method proposed by Tsai and Lenz (1989) was used to estimate the unknown matrix *X*, and the matrix was also called the extrinsic matrix of the 3D scanner.

### 3.3. Global Optimization Strategy

In the previous sections, we have estimated the desired matrix *X*. However, given the positioning error of the tracker and the pointset registration error, the calibration result may shift toward a specific position and orientation. To solve this problem, we proposed a global optimization strategy to fine-tune matrix *X*. We designed a cost function, and the matrix *X* obtained in section 3.2 was used as the initial value. Then, matrix *X* was fine-tuned through the Levenberg-Marquart (Levenberg, 1944) nonlinear optimization algorithm.

To make the extrinsic matrix X globally optimal, the designed cost function used must be able to calculate the distance between multiple pointsets scanned in the hand-eye calibration step (section 3.2). Therefore, we need to extend the commonly used distance measure between two pointsets to the calculation of multiple pointsets. Assume that *PTS*_*i*_(*i* = 0, ⋯ , *n*) are collected pointsets in different positions and orientations, where *n* represents the number of collected pointsets. At the same time, the corresponding transformation matrix of the optical marker to the world coordinate system is ${}_{Marker}^{\;NDI}T_{i}$. Then, the transformation of the *i*-th pointset from the scanner coordinate system to the world coordinate system can then be expressed as

(6) $$\begin{array}{l} {}_{Scanner}^{\;NDI}T_{i}={}_{Marker}^{\;NDI}T_{i}{}_{Scanner}^{\;Marker}T_{i} \end{array}$$

Using this matrix, we could unify all pointsets into the world coordinate system as follows

(7) $$\begin{array}{l} PTS_{i}^{\prime }={}_{Scanner}^{\;NDI}T_{i}PTS_{i}\left(i=0,\cdots \;,n\right) \end{array}$$

Given a distance constraint metric δ (δ = 5*mm* in this study), the alignment error between two pointsets was defined as the root mean square that meet the distance constraints metric, which can be expressed as

(8) $$\begin{array}{l} RMS\left(P,Q\right)=\sqrt{\frac{1}{n}\overset{n}{\underset{i=1}{\sum }}\|T_{trans}p_{i}-q_{i}{\|}^{2}} \end{array}$$

where *T*_*trans*_ is the transformation matrix between two pointsets; *p*_*i*_ and *q*_*i*_ are the *i*-th point in *P* and *Q*, respectively; and *n* is the number of points that meet the distance constraint metric δ. The alignment error between a pointset and the other pointsets is then defined as

(9) $$\begin{array}{l} RMS_{i}=\frac{1}{n-1}\overset{n}{\underset{j=0,j\neq i}{\sum }}RMS\left(PTS_{i}^{\prime },PTS_{j}^{\prime }\right) \end{array}$$

where *n* is the number of collected pointsets. Therefore, the object of nonlinear optimization is defined as minimizing the average registration error of all pointsets called repeat scan registration error (RSRE), which is formulated as

(10) $$\begin{array}{l} RSRE\left(R,t\right)=\underset{\left(R,t\right)}{\arg\min}\frac{1}{n}\overset{n}{\underset{i=0}{\sum }}RMS_{i} \end{array}$$

As shown in Equation (10), RSRE is a measure used to quantify the average spatial distance between multiple pointsets. By taking RSRE as the cost function of nonlinear optimization, the extrinsic matrix *X* initially obtained was fine-tuned. In this manner, the ultimately obtained extrinsic parameters of the 3D scanner are globally optimal and thus the error distribution of the scanned pointsets in space becomes more uniform, which is similar to the bundle adjustment algorithm (Triggs et al., 2000; Jeong et al., 2012; Liu et al., 2018).

### 3.4. Application of the 3D Scanner in Image-To-Patient Registration

**Figure 5:**
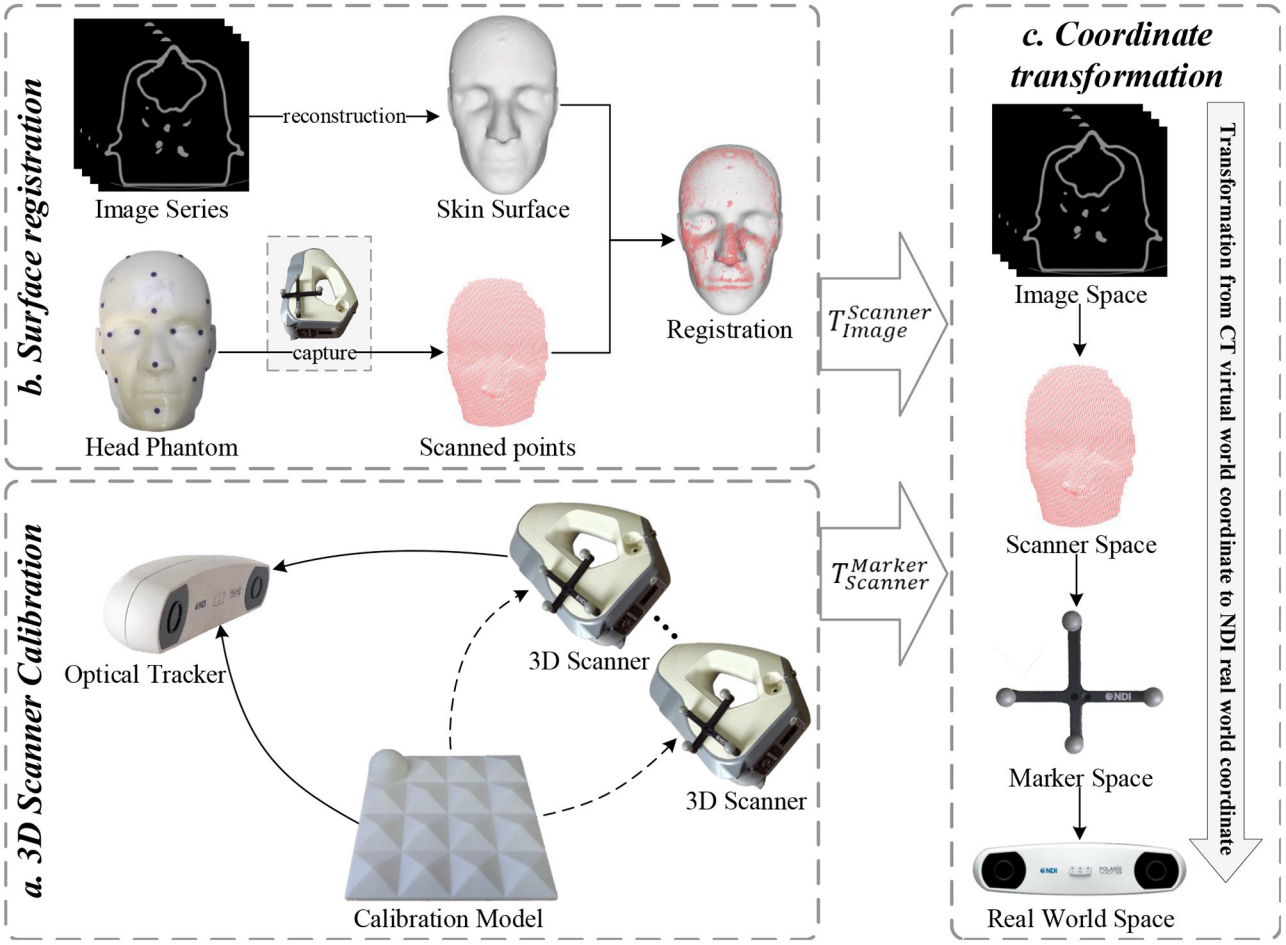
Flowchart of the proposed 3D scanner-based image-to-patient registration method.

This section describes the application of the 3D scanner in image-to-patient registration. Several experiments were designed to verify the superiority of the proposed calibration method. We constructed a surgical navigation system based on an optically tracked 3D scanner to perform image-to-patient registration. Figure 5 shows the flowchart of the proposed registration method, which is mainly divided into three parts, namely, 3D scanner calibration, surface registration, and coordinate transformation.

First, the 3D scanner was calibrated using the proposed two-step calibration method, and the transformation matrix $T_{Scanner}^{Marker}$ between the 3D scanner and optical marker was estimated. This step is usually performed in an experimental environment. Second, by matching the pointsets collected by the 3D scanner with the pointsets reconstructed from the medical images of a patient, these medical images could be transformed into the coordinate system of the 3D scanner. Third, the tracker detected and determined the position and orientation of the optical marker attached to the 3D scanner in real time. Combined with the calibrated extrinsic matrix $T_{Scanner}^{Marker}$ of the 3D scanner, the medical images could be transformed into the world coordinate system where the patient is located, thereby completing the image-to-patient registration process.

Pointset registration presents a fundamental problem in computer vision. The well-known ICP algorithm is widely used in the rigid registration of pointsets given its high efficiency and good performance. However, ICP is also known for its tendency to fall into the local minima. Therefore, convergence can only be guaranteed when the pointsets to be registered are roughly aligned. Furthermore, the ICP algorithm performs poorly when addressing the pointset registration problem that involves small coverage or large differences in poses. Therefore, an initial pose transformation should be performed to roughly align the two pointsets, and the ICP algorithm should be used for fine registration.

The scanner-based image-to-patient registration method faces two problems, that is, (1) the uncertainty of the posture of a patient in the navigation space creates a huge difference in the initial posture of the pointsets to be registered; and (2) a pointset containing the entire face of the patient cannot be captured due to the limitations of the surgical environment, and the captured pointsets may contain a large percentage of outliers. To address the first problem, a coarse registration of the pointset extracted from medical images using the captured pointset must be performed by manually selecting paired points (Fan et al., 2014). However, doing so requires considerable manual interaction, thereby increasing the complexity of the operation. Therefore, a coarse registration method that ignores the initial pose of the pointsets must be developed. The second problem is a typical local registration problem of pointsets. For the clinical scenario, the captured facial pointsets of the patient may only partially overlap the pointsets extracted from medical images. Therefore, the largest common pointset should be used as the similarity measure in coarse registration.

In this study, the coarse-to-fine registration strategy was used for the pointset registration process. Coarse registration was achieved using the Super4PCS algorithm proposed by Mellado et al. (2015) to obtain a good initial posture between two pointsets. Afterward, the ICP algorithm was used to achieve a fine registration and find the *R* (rotation matrix) and *t* (translation vector) that best align two pointsets. Let *P* = *p*_1_, *p*_2_, ⋯ , *p*_*m*_ and *Q* = *q*_1_, *q*_2_, ⋯ , *q*_*n*_ be the facial pointset captured by the 3D scanner and the corresponding facial pointset extracted from the medical image, respectively. The registration goal of these two pointsets is to minimize the following matching error:

(11) $$\begin{array}{l} E\left(R,t\right)=\underset{\left(R,t\right)}{\arg\min}\overset{N}{\underset{i=1}{\sum }}\|\left(R\left(T_{Marker}^{NDI}T_{Scanner}^{Marker}p_{i}\right)+t\right)-q_{i}\| \end{array}$$

where $T_{Scanner}^{Marker}$ is the extrinsic matrix calibrated by the proposed two-step calibration method, and $T_{Marker}^{NDI}$ is the transformation matrix of the optical marker attached to the 3D scanner that is tracked by the optical tracker in real time. Therefore, $T_{Marker}^{NDI}T_{Scanner}^{Marker}p_{i}$ could be used to represent the pointsets captured by the 3D scanner after being transformed into the world coordinate system. A relationship between the virtual-world coordinate system located by CT and the real-world coordinate system located by the patient was then established, thereby completing the image-to-patient registration process.

## 4. Experiments and Results

A series of 3D models were designed to evaluate the superiority of the proposed method. Afterward, the feasibility of the proposed 3D scanner-based image-to-patient registration method was evaluated. The influence of the residual error of the extrinsic matrix of the 3D scanner on TRE was then evaluated, and the importance of accurately calibrating the extrinsic parameters of the 3D scanner was highlighted.

### 4.1. Evaluation of Different Calibration Methods

**Figure 6:**
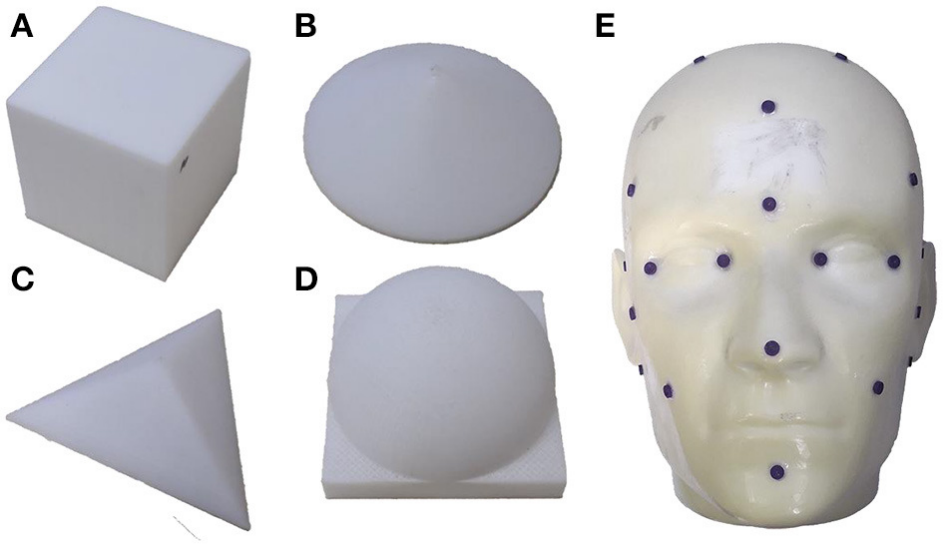
3D models used in the experiments. Regular models: **(A)** cube; **(B)** cone; **(C)** tetrahedron; and **(D)** hemisphere. Irregular model: **(E)** head phantom.

The performance evaluation experiments were divided into two parts. As shown in Figure 6, the regular models include a plane (the upper plane of a cube with a side length l = 60 mm), a cone (bottom radius r = 30 mm, height h = 20 mm), a tetrahedron (side length l = 60 mm), and a hemisphere (radius r = 30 mm). The irregular model was a head phantom whose size is the same as that of a real human head.

The model surfaces were collected by the optically tracked 3D scanner in different positions and orientations and were transformed into the world coordinate system using the calibrated extrinsic matrix of the 3D scanner. Given that we could not obtain the ground truth of the extrinsic matrix, the designed error model RSRE and Chamfer distance (CD) were used to evaluate the performance of the calibration method. CD was first introduced by Hilditch (1969) and studied by Borgefors (1984) to approximate the Euclidean metric. It has also been widely used to measure the similarity between pointsets in point cloud registration and reconstruction tasks (Wu et al., 2015; Fan H. et al., 2017; Jiang et al., 2018). Suppose two pointsets *P* and *Q* have *n*_*P*_ and *n*_*Q*_ points respectively. Then, the CD between them is defined as:

(12) $$\begin{array}{l} CD\left(P,Q\right)=\frac{1}{n_{P}}\underset{p\in P}{\sum }\underset{q\in Q}{\min}\|p-q{\|}_{2}^{2}+\frac{1}{n_{Q}}\underset{q\in Q}{\sum }\underset{p\in P}{\min}\|p-q{\|}_{2}^{2} \end{array}$$

The distance metric was set to δ = 5*mm*, and the following calibration methods were compared:

1. Pick-2D: Obtains the closed-form solution of the extrinsic matrix based on the 2D checkerboard model calibrated by the RGB images and the points picked by the optically tracked pointer;
2. Hand-Eye-2D: Obtains the numerical solution of the extrinsic matrix based on the 2D checkerboard model calibrated by the RGB images and its mapping relationship with the depth image;
3. Hand-Eye-3D: Proposed without global optimization; and
4. Proposed method: As described above.

The surfaces of the models were collected at seven positions, including directly above and around the model, as a verification dataset. The experimental results are shown in Table 1.

**Table 1:** Comparison of different calibration methods.

**Methods**	**Plane (mm)**		**Cone (mm)**		**Tetrahedron (mm)**		**Hemisphere (mm)**		**Phantom (mm)**	
	**RSRE**	**CD**	**RSRE**	**CD**	**RSRE**	**CD**	**RSRE**	**CD**	**RSRE**	**CD**
Pick-2D	1.07	1.19	1.81	3.17	2.32	5.40	2.36	4.75	2.10	3.98
Hand-Eye-2D	0.95	0.98	1.67	3.17	2.26	5.39	2.14	4.38	1.94	3.46
Hand-Eye-3D	0.99	1.03	1.63	2.81	2.03	4.29	1.72	2.90	1.83	3.17
Proposed	0.95	0.92	1.34	1.92	1.68	2.92	1.72	2.88	1.73	2.83

The verification experiment results reveal that the proposed calibration method has a significantly higher accuracy than the other methods. The Pick-2D has the largest calibration error largely due to the point selection error introduced by manual participation. The calibration error of the Hand-Eye-2D is slightly smaller than the closed-form solution obtained by the Pick-2D. The Hand-Eye-3D method has a smaller calibration error than the other methods. Nevertheless, after the global optimization process, the proposed method has achieved improvements in accuracy and outperformed all the other methods.

**Figure 7:**
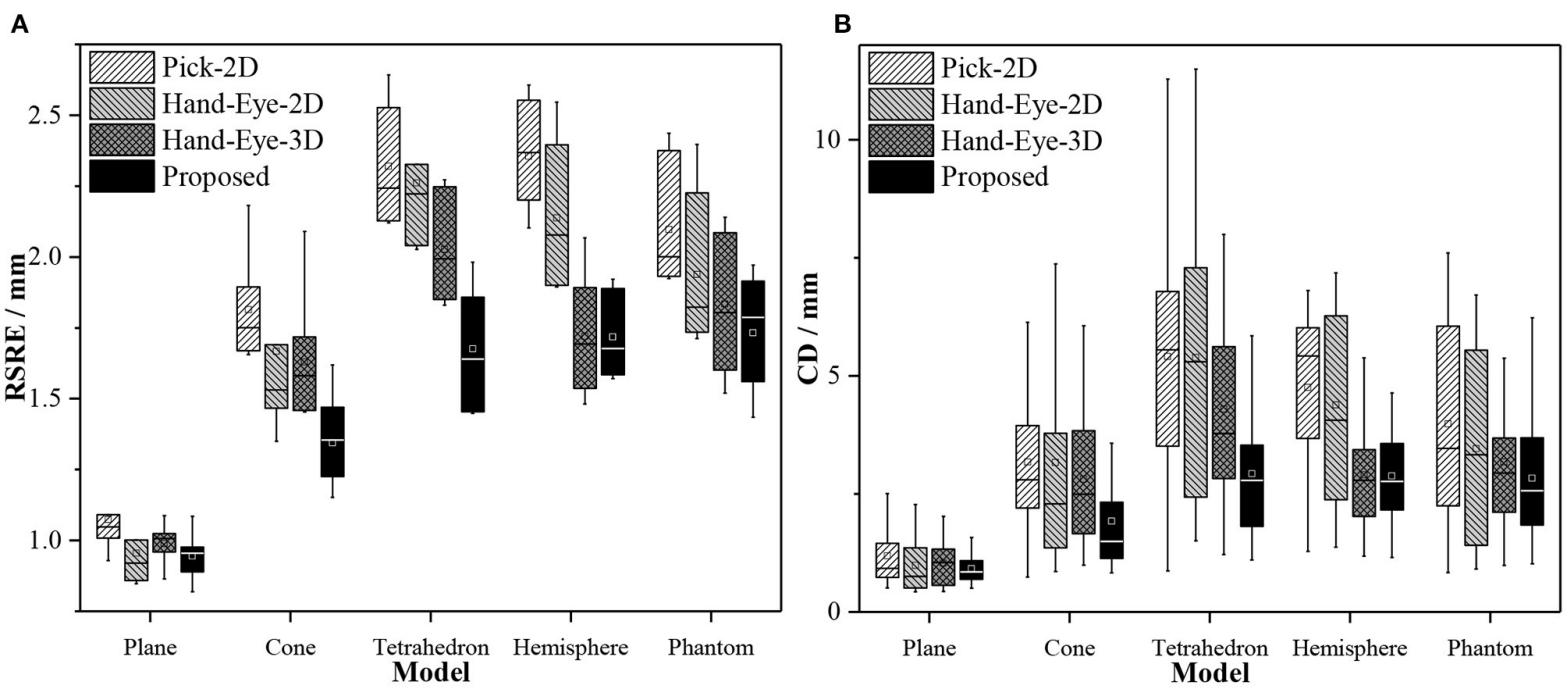
Box plots of the accuracy comparison among different calibration methods. **(A)** RSRE and **(B)** CD.

Figure 7 compares the performance of the proposed method with that of the Pick-2D, Hand-Eye-2D, and Hand-Eye-3D. These box plots show the error distribution among different methods under multiple independently repeated experiments. In the experiment that used the simplest plane model, the space complexity of the model is low, and the errors of all methods were similarly low. As the complexity of the model increases, its ability to describe the 3D space also improves, whereas the error level gradually increases. Obviously, the RSRE and CD errors of the proposed method are always the smallest.

**Figure 8:**
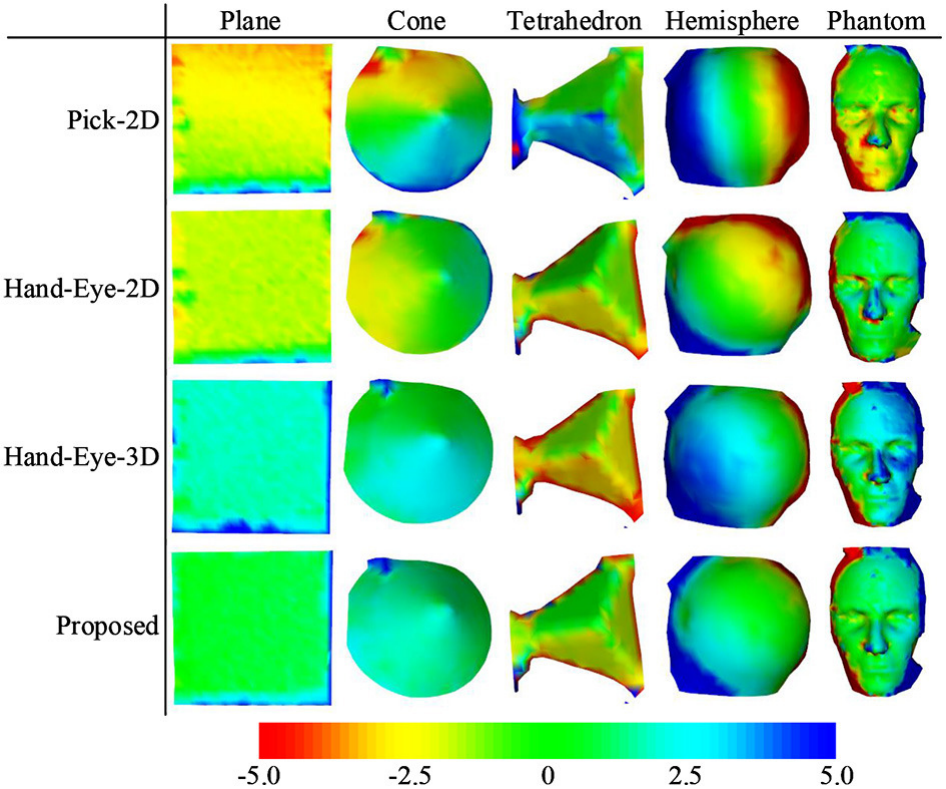
Distance maps calculated by different calibration methods (with the first pointset as the matching reference).

Figure 8 shows the distance maps between the first pointset (fixed directly above the model) and the rest of the random pointset in the verification dataset. The distance map directly shows the errors in different areas between the two pointsets. Green means that the error is zero, and the error increases as the color changes to red or blue. As shown in Figure 8, a red or blue area appears in each graph, indicating that a large error occurs in this area. By contrast, the large errors in the distance map related to the proposed method are mostly concentrated in the edge area, and this area is relatively smaller than the entire graph. The estimated maximum error is <2.5 units.

### 4.2. Effects of Different Calibration Methods on TRE

**Figure 9:**
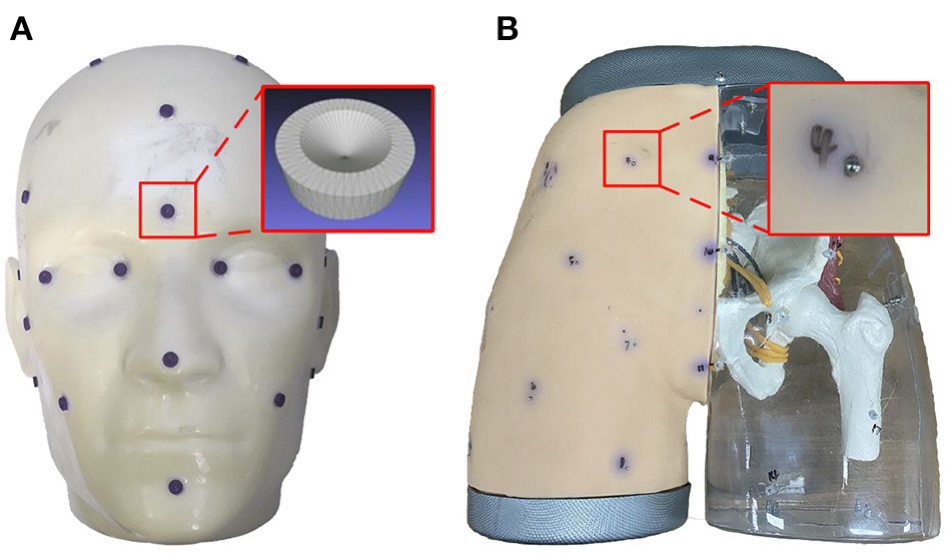
Models designed for the experiments: **(A)** head phantom; and **(B)** hip phantom.

To further evaluate the superiority of the proposed calibration method, we constructed a surgical navigation system and used the calibrated 3D scanner to perform image-to-patient registration. TRE was then used to measure the positioning accuracy of the surgical navigation system. The feasibility of 3D scanner-based image-to-patient registration was also validated. As shown in Figure 9, a head phantom attached with 21 designed fiducial points and a hip phantom attached with 19 steel balls (d = 1 mm) were used to complete the experiments.

**Figure 10:**
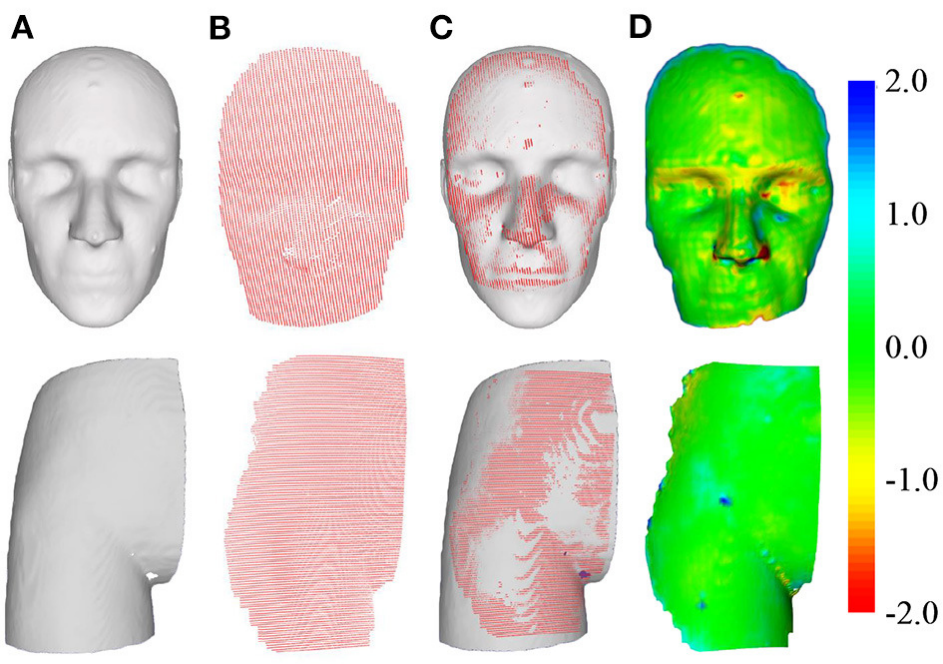
Schematic of pointset registration. The top row is the head phantom, and the bottom row is the hip phantom. **(A)** Surface of the phantom extracted from CT. **(B)** Surface of the phantom captured by the 3D scanner. **(C)** Pointset overlay diagram after registration. **(D)** Distance map of the overlapped area after registration.

The designed phantom was scanned and reconstructed by thin-slice CT with a thickness of 0.625 mm, and the marching cubes algorithm (He, 1987) was used to extract the surface of the phantom in the image for subsequent registration. Multiple sets of independently repeated experiments were performed, and the data obtained from each experiment included (1) *PTS*_*source*_: the skin pointset of the phantom extracted from the CT image; (2) *PTS*_*target*_: the skin pointset of the phantom captured by the 3D scanner; (3) $T_{Marker}^{NDI}$: the spatial transformation matrix to the world coordinate system of the optical marker fixed on the 3D scanner; (4) $P_{virtual}^{i}$: the fiducials picked from the CT image of the phantom; and (5) $P_{real}^{i}$: the fiducials picked from the real world by the optically tracked pointer. The point index in the fiducial points is represented by *i*. The captured pointsets were transformed into the world coordinate system and can be expressed as

(13) $$\begin{array}{l} PTS_{target}^{NDI}=T_{Marker}^{NDI}T_{Scanner}^{Marker}PTS_{target} \end{array}$$

where $T_{Scanner}^{Marker}$ is the extrinsic matrix of the 3D scanner. The pointset registration process was performed to align pointset *PTS*_*source*_ with $PTS_{target}^{NDI}$, and the transformation matrix $T_{Image}^{NDI}$ from the virtual coordinate system of CT to the real-world coordinate system could be obtained. Then, the final positioning error TRE can be expressed as

(14) $$\begin{array}{l} TRE=\frac{1}{n}\overset{n}{\underset{i=1}{\sum }}\|T_{Image}^{NDI}P_{virtual}^{i}-P_{real}^{i}\| \end{array}$$

where *n* represents the number of fiducial points used to evaluate errors, and the operator ||·|| represents the Euclidean distance between two points in the Euclidean space. Figure 10 shows the schematic of the registration of the two pointsets. The distance map reveals that after registration, except for the extremely few red and blue areas (e.g., the edges of the phantom) and other areas due to incomplete scanning, the rest are almost all green and yellow. In other words, the maximum error of registration is controlled below 1 unit.

We performed seven sets of independently repeated experiments and separately conducted simulation experiments using Pick-2D, Hand-Eye-2D, Hand-Eye-3D, and the proposed method to obtain the extrinsic matrix of the 3D scanner. Table 2 shows the experimental results. The TRE values calculated by Pick-2D, Hand-Eye-2D, and Hand-Eye-3D are all relatively high, whereas that obtained by the proposed method is more accurate. In the experiment using head phantom, the TRE is controlled within 2 mm, except for one set where accuracy has reached 2.15 mm. In the experiment using hip phantom, the TRE is relatively high overall, which may be caused by the lack of 3D features of phantom affecting the registration result of pointsets.

**Table 2:** TRE values obtained by different methods.

**Trials**	**Head phantom (mm)**				**Hip phantom (mm)**			
	**Pick-2D**	**Hand-Eye-2D**	**Hand-Eye-3D**	**Proposed**	**Pick-2D**	**Hand-Eye-2D**	**Hand-Eye-3D**	**Proposed**
#1	5.69	4.08	2.61	1.66	7.66	5.73	3.61	2.58
#2	5.33	4.70	3.56	1.77	7.94	5.37	2.54	2.02
#3	6.88	3.54	3.16	2.15	7.52	6.14	4.41	2.77
#4	4.27	6.26	4.23	1.87	7.40	6.27	4.81	3.59
#5	5.21	4.00	3.27	1.82	7.77	5.86	3.51	2.27
#6	4.66	5.13	3.86	1.64	7.64	6.26	5.01	2.73
#7	4.98	4.85	3.09	1.63	6.83	5.58	4.44	3.17
Mean	5.29	4.65	3.40	1.79	7.54	5.89	4.05	2.73
Std	0.78	0.83	0.50	0.17	0.33	0.33	0.81	0.49

**Figure 11:**
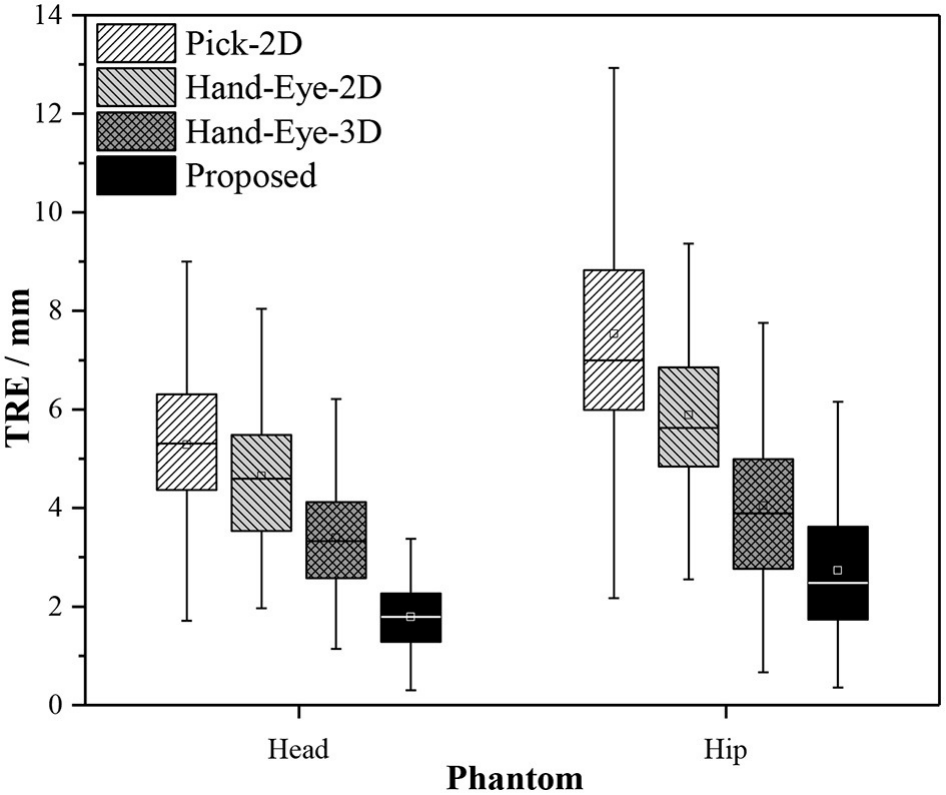
Box plots of the experiment results obtained by different calibration methods used in image-to-patient registration.

Figure 11 compares the performance of the proposed method with those of Pick-2D, Hand-Eye-2D, and Hand-Eye-3D. In the seven sets of independently repeated experiments using two phantoms, the proposed method shows obvious superiority over the other three methods.

### 4.3. Effect of Residual Transformation Noise on TRE

In section 4.2, a comparison of several methods reveals that when the RSRE and CD of the calibration result is only slightly different (RSRE and CD achieve 1.73–2.10 and 2.83–3.98 mm in the head phantom experiment, respectively), the TRE values greatly vary between 1.79 and 5.29 mm. Therefore, we further verified the influence of the residual errors of the rotation and translation components in the extrinsic matrix on the final positioning accuracy by performing simulation experiments. Using the experimental results in section 4.2 as reference, we added different levels of noise to the matrix $T_{Scanner}^{Marker}$ in Equation (11) for the simulation experiments.

The experiment was divided into two parts. First, we decomposed the rotation matrix into rotation angle components along three coordinate axes, added degree noise at 0.1° intervals from −0.5 to 0.5° to each of the three rotation angle components, and calculated the final TRE value. We used the same method to add translation noise at 0.5 mm intervals from −2.5 to 2.5 mm to the translation components on the three coordinates axes and calculated the final TRE value. Table 3 shows the results.

**Table 3:** Results of residual noise verification.

**Noise (degree)**	**−0.5**	**−0.4**	**−0.3**	**−0.2**	**−0.1**	**0.0**	**0.1**	**0.2**	**0.3**	**0.4**	**0.5**
Mean (mm)	4.84	3.72	2.66	1.74	1.30	1.66	2.60	3.68	4.80	5.94	7.09
Std (mm)	1.05	0.92	0.79	0.63	0.48	0.68	0.72	0.76	0.82	0.91	1.02
Noise (mm)	−2.50	−2.00	−1.50	−1.00	−0.50	0.00	0.50	1.00	1.50	2.00	2.50
Mean (mm)	5.32	4.49	3.68	2.90	2.19	1.66	1.53	1.79	2.34	3.05	3.83
Std (mm)	0.76	0.77	0.77	0.77	0.75	0.68	0.47	0.50	0.58	0.61	0.64

**Figure 12:**
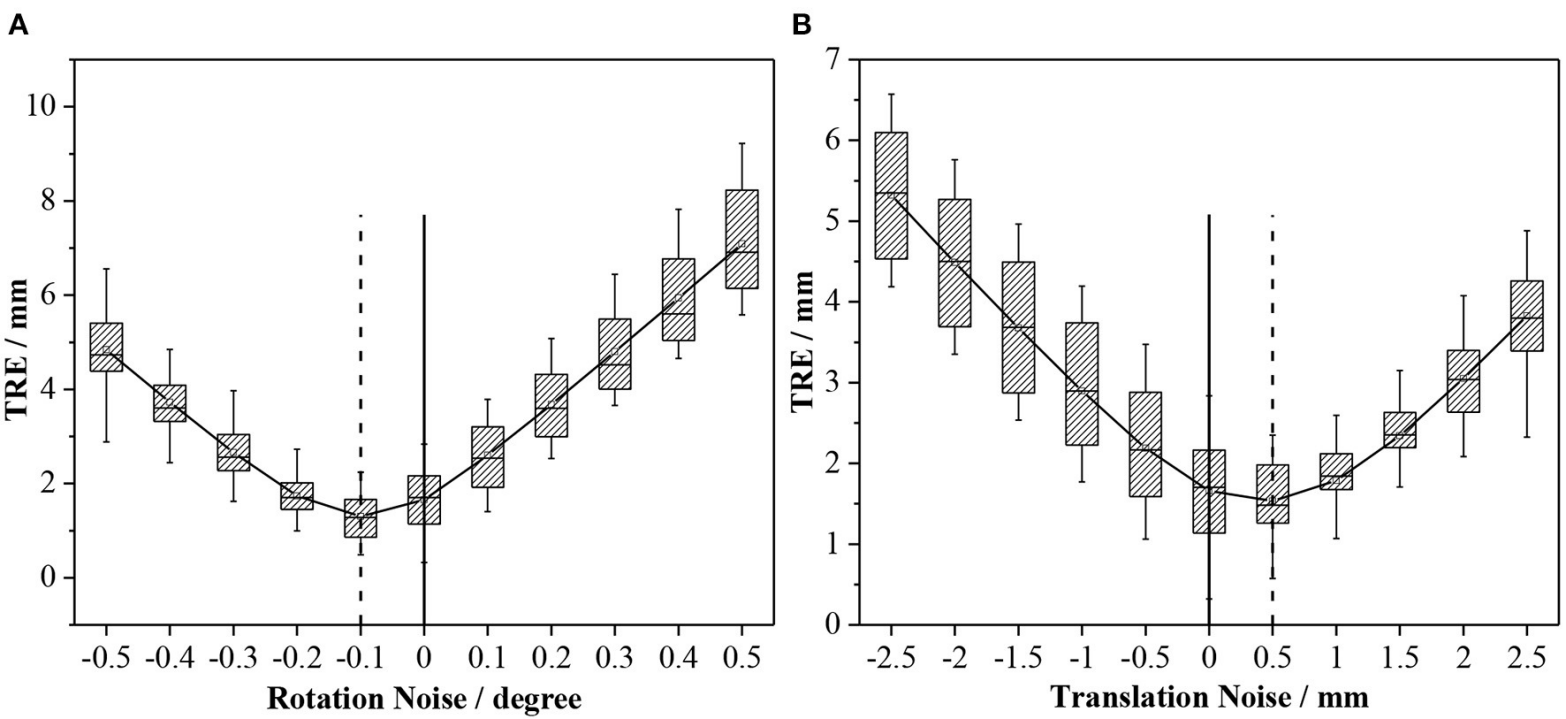
Effects of different levels of noise on TRE: **(A)** Rotation noise; and **(B)** translation noise.

Figure 12 shows the effect of residual noise, including rotation and translation noises, on TRE. As the amount of added noise increases, the final mean and variance of TRE generally show an upward trend. At the same time, the TRE decreases after adding −0.1° rotation noise or 0.5 mm translation noise (dotted line). This abnormal phenomenon is often caused by residual errors in the calibration matrix or the pointset registration process. Moreover, every small angle (0.1°) or translation (0.5 mm) of the extrinsic matrix can cause a large change (0–2 mm) in the final TRE value. In other words, the calibration accuracy of the 3D scanner has a vital influence on the accuracy of subsequent applications.

**Figure 13:**
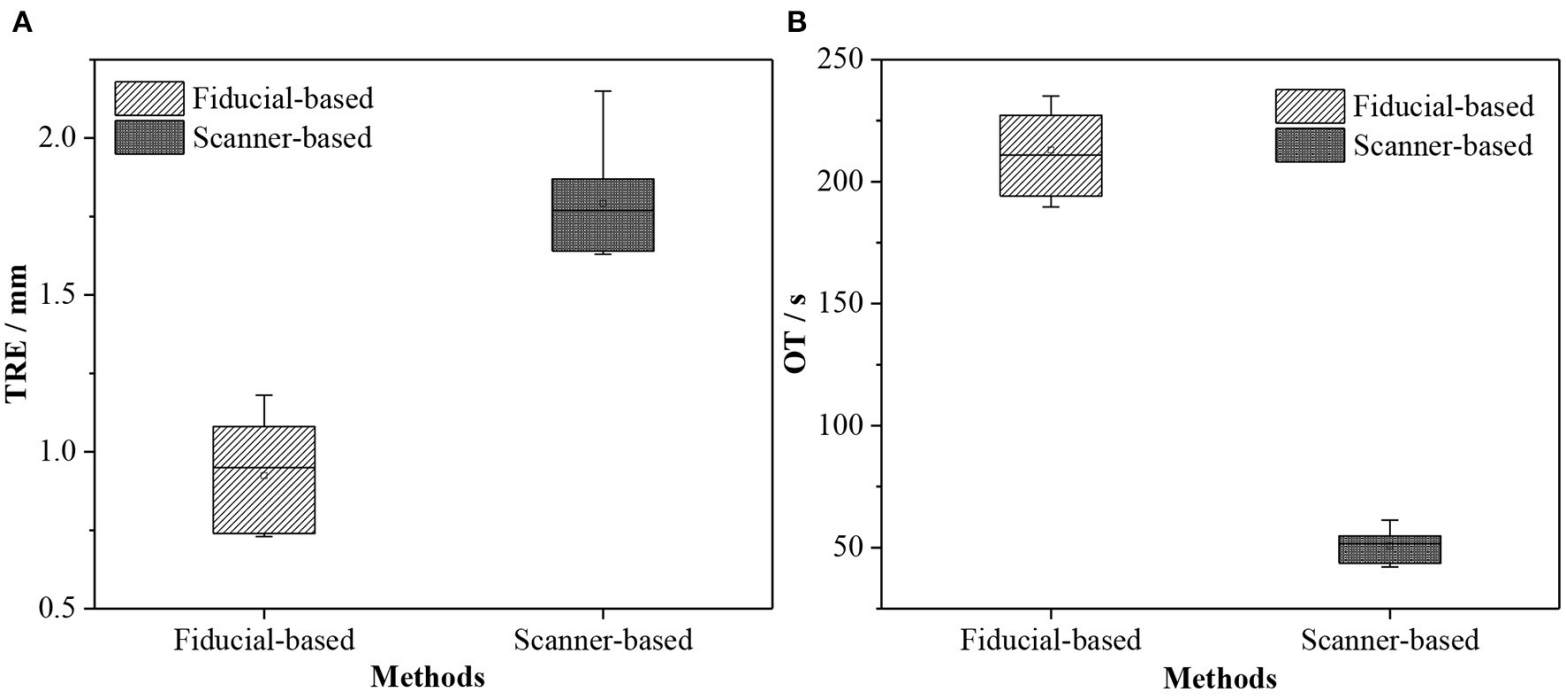
Box plots of TRE and OT obtained by different registration methods. **(A)** TRE and **(B)** OT.

## 5. Discussion

We proposed a method for accurately calibrating the extrinsic parameters of an optically tracked 3D scanner based on pointset registration and nonlinear optimization technique. In the image-to-patient registration experiments, the proposed calibration method achieved the best accuracy results. However, after analyzing previous research on image-to-patient registration (Gerber et al., 2013; Soteriou et al., 2016; Perwg et al., 2018), we found that the TRE value achieved by the proposed 3D scanner-based method was not the best.

Gerber et al. (2013) and Chu et al. (2017) argued that the image-to-patient registration based on artificial fiducials is highly accurate and can even reach submillimeter navigation accuracy. Therefore, we compared our proposed image-to-patient registration method with the artificial fiducial-based method in two aspects, namely, TRE and operation time (OT). We selected five noncoplanar fiducials to perform fiducial-based registration, and the OT included selecting fiducials from the image, picking fiducials in the real world, and running the fiducial point registration algorithm. Moreover, the OT of the scanner-based method included extracting skin from the image, collecting the pointset of the model using the 3D scanner, and running the pointset registration algorithm.

According to Fitzpatrick et al. (2002), TRE refers to the distance between the position of the fiducial that is not used for the registration and the corresponding position in the real-world coordinate system after registration. Given that we applied pointset registration, all identification points deviated from the skin surface and were not used for registration. Therefore, we used those fiducial points that were not used for registration to evaluate the accuracy of fiducial-based registration and then used all fiducial points to evaluate the accuracy of the proposed registration method. We conducted seven independently repeated experiments on the head phantom and evaluated the errors of 21 fiducials on the phantom surface. Table 4 presents the experiment results.

**Table 4:** Results of different registration methods.

**Trials**	**Fiducial-based**		**Scanner-based**	
	**TRE (mm)**	**OT (s)**	**TRE (mm)**	**OT (s)**
#1	0.95	225.27	1.66	42.11
#2	1.18	194.05	1.77	54.82
#3	0.99	208.48	2.15	51.71
#4	0.73	210.94	1.87	43.55
#5	0.80	235.12	1.82	50.02
#6	0.74	227.24	1.64	51.57
#7	1.08	189.66	1.63	61.26
Mean	0.92	212.97	1.79	50.72
Std	0.16	15.91	0.17	6.04

The accuracy of the proposed 3D scanner-based registration method was 1.79±0.17 mm. Although this method could meet the clinical navigation requirements (<2 mm), its accuracy was worse than that of the fiducial-based method (0.92±0.16 mm) due to the residual error in the extrinsic matrix of the scanner or the registration matrix of the pointsets. We have also verified this finding in section 4.3. The proposed 3D scanner-based registration method also had higher time efficiency than the fiducial-based method (50.72±6.04*s* vs. 212.97±15.91*s*), thereby saving valuable time for clinicians and patients.

The proposed 3D scanner-based image-to-patient registration method also does not require fiducials to be pre-attached on the face of patients before performing a CT scan, thereby simplifying the operation process to a certain extent and reducing the surgical costs. This method also does not require contact with the skin of patients, thereby guaranteeing operation safety. Therefore, the 3D scanner-based image-to-patient registration method is exceptionally friendly to the clinical environment, but its accuracy warrants improvement before its application in image-to-patient registration.

## 6. Conclusion

Tracked 3D scanners are increasingly being used in IGS. External tracking markers usually need to be bound with the 3D scanner, and the 3D scanner can then be tracked in real time. In this manner, the geometric data captured by the 3D scanner can be aligned with the medical images of patients to achieve a precise positioning of their anatomical structures or tissues. Therefore, a precise calibration between the 3D scanner and the external tracker is particularly important. However, this problem has not yet been investigated in detail.

The 3D scanner captures RGB and depth images and performs texture mapping through the projection matrix between RGB and depth cameras. We can obtain the depth image or pointset with texture. Given that RGB and depth cameras are independent, some residual errors may be observed in the coordinate mapping between them. Therefore, calibrating the 3D scanner using an RGB image will introduce unnecessary errors and render the results inaccurate.

To address these issues, we proposed a novel method for calibrating the extrinsic parameters of the 3D scanner. First, the surface of the benchmark was captured by the tracked 3D scanner in multiple positions and orientations. A series of equations were then formulated using the pointset registration technique and the coordinate transformation process to estimate the initial extrinsic matrix of the 3D scanner. Second, an error model called RSRE was constructed and used as the cost function of the nonlinear optimization algorithm to obtain the global optimal extrinsic matrix. Experimental results show that the proposed calibration method has a lower RSRE and CD value than the others based on 2D checkerboard.

Third, we constructed a surgical navigation system based on an optically tracked 3D scanner. On the one hand, the comparison of the influence of the extrinsic matrix of the 3D scanner obtained by different calibration methods on TRE indicates that the proposed calibration method obtains the lowest TRE value. On the other hand, in comparison with the fiducial-based image-to-patient registration method, the efficiency of the proposed method is greatly improved. Although its accuracy is not as good as that of the fiducial-based registration method, the proposed 3D scanner-based registration method still meets the clinical requirements and demonstrates noncontact and high safety benefits, thereby highlighting its significant research value.

When using scanner-based registration method, data should be subjected to multiple spatial transformations. In this case, a small residual error may lead to huge errors in the final transformation. Given the limited space and complex clinical environment in operating rooms, problems such as an incomplete acquisition of the facial pointset of the patient and a low pointset registration accuracy may restrict the application of scanner-based image-to-patient registration. We aim to address these problems by conducting a follow-up work and verifying our findings in the clinical scenario.

## Data Availability Statement

The original contributions presented in the study are included in the article/supplementary material, further inquiries can be directed to the corresponding author/s.

## Author Contributions

WL, JF, SL, ZT, DA, HS, and JY: conception and design of study. WL, JF, and SL: analysis and interpretation of data. WL, ZT, and ZZ: acquisition of data. All authors contributed to the article and approved the submitted version.

## Conflict of Interest

ZT was employed by company Ariemedi Medical Technology (Beijing) CO., LTD. The remaining authors declare that the research was conducted in the absence of any commercial or financial relationships that could be construed as a potential conflict of interest.
